# Phytochemicals in Drug Discovery—A Confluence of Tradition and Innovation

**DOI:** 10.3390/ijms25168792

**Published:** 2024-08-13

**Authors:** Patience Chihomvu, A. Ganesan, Simon Gibbons, Kevin Woollard, Martin A. Hayes

**Affiliations:** 1 Compound Synthesis and Management, Discovery Sciences, Biopharmaceuticals R&D, AstraZeneca, 431 83 Mölndal, Sweden; 2 School of Chemistry, Pharmacy & Pharmacology, University of East Anglia, Norwich Research Park, Norwich NR4 7TJ, UK; a.ganesan@uea.ac.uk; 3 Natural and Medical Sciences Research Center, University of Nizwa, Birkat Al Mawz 616, Oman; simon@unizwa.edu.om; 4 Bioscience Renal, Research and Early Development, Cardiovascular, Renal and Metabolic, BioPharmaceuticals R&D, AstraZeneca, Cambridge CB21 6GH, UK; kevin.woollard@astrazeneca.com

**Keywords:** phytochemicals, natural products, traditional medicine

## Abstract

Phytochemicals have a long and successful history in drug discovery. With recent advancements in analytical techniques and methodologies, discovering bioactive leads from natural compounds has become easier. Computational techniques like molecular docking, QSAR modelling and machine learning, and network pharmacology are among the most promising new tools that allow researchers to make predictions concerning natural products’ potential targets, thereby guiding experimental validation efforts. Additionally, approaches like LC-MS or LC-NMR speed up compound identification by streamlining analytical processes. Integrating structural and computational biology aids in lead identification, thus providing invaluable information to understand how phytochemicals interact with potential targets in the body. An emerging computational approach is machine learning involving QSAR modelling and deep neural networks that interrelate phytochemical properties with diverse physiological activities such as antimicrobial or anticancer effects.

## 1. Introduction

Phytochemicals are plant natural products that possess numerous therapeutic properties. Traditional medicines have utilised the beneficial properties associated with these compounds for centuries, highlighting their potential to become novel drug candidates [1]. Modern scientific approaches such as structural and computational biology offer unprecedented opportunities to study these natural products further. Analysis conducted via structural biology techniques has revealed three-dimensional structures of phytochemicals that can aid investigations with molecular docking or virtual screening to find new pharmacologically active molecules [2]. This review will explore the role of phytochemicals in modern-day drug discovery. We highlight essential findings and trends in this field from 1995, focusing on the methodologies used, the challenges encountered, and future research prospects.

### 1.1. The Role of Phytochemicals in Traditional Medicine

Traditional medicine encompasses approaches from indigenous knowledge systems and is heavily influenced by phytochemicals derived from plants that have both cultural and historical significance [3]. Plant-based remedies have shaped modern pharmacology through the identification of valuable therapeutic agents present within them. With its interdisciplinary approach, ethnopharmacology seeks to understand how these specific phytochemicals function as part of traditional healing methods [1].

Approximately 70−80% of the global population uses traditional medicines to treat diseases [4]. For those living in rural regions across the globe with limited access to advanced Western medical practices and technology, traditional medications continue to be an essential aspect of inclusive healthcare solutions [5]. Phytomedicines are derived from centuries-old healing traditions from the natural resources surrounding them. Moreover, these medicines often emphasise curing disease beyond the physical symptoms alone; they maintain a patient’s overall psychological and ethical balance as integral determinants of health, making phytomedicines beneficial amongst community members who value holistic recovery [6]. Many such preparations are extracted directly from plants; some can be modified by structural transformation. Given the natural diversity of plants globally, there are many unidentified phytochemicals whose biological actions are yet to be discovered [7]

In recent years, traditional Chinese and African medicines have been used against diseases, including COVID-19. For instance, the National Administration of Traditional Chinese Medicine organised a study to identify potential treatments against COVID-19, and the Lung Cleansing and Detoxifying Decoction (LCDD) was widely used and studied through clinical trials. LCDD contains 21 ingredients, including *Ephedra sinica*, *Atractylodes macrocephala*, and *Scutellaria baicalensis*, which likely counteract COVID-19 through synergistic activities [8]. The first trial showed that LCDD was effective on 90% of the 214 COVID-19 patients. Further trials were carried out on a more extensive trial group with 1262 patients, including 57 with severe symptoms. The results showed that 99.28% of the patients recovered, and none developed severe symptoms during the treatment [8]. In Africa, an elixir based on *Artemisia annua* extract, known as “covid-organics”, was used as a potential cure for COVID-19, and studies are still ongoing [9]. Plant-based antimalarials like artemisinin from *Artemisia* spp. have also been tested against the SARS-CoV-2 virus [10].

*Panax ginseng* has been widely used as a healing plant in Asian traditional medicine. This species contains many natural products, including ginsenosides, that exert qualities such as improving immune health, reducing inflammation, and having anticancer effects [11]. Similarly, turmeric, or *Curcuma longa*, a plant in the ginger family *Zingiberaceae*, which is prominent in Ayurvedic culture, contains curcumin and has wound-healing abilities and antioxidant and anti-inflammatory activities [12]. Moreover, *Echinacea* from North America is effective against respiratory disorders [13]. *Echinacea* possesses phytochemicals such as phenolics, including caftaric acid, chicoric acid, cynarin, chlorogenic acid, and echinacoside. Volatile terpenes, such as germacrene D and polyacetylene, are also present and possess antimicrobial and antioxidant activities. Ascorbic acid is also present, and it aids in immune augmentation. The polysaccharides and glycoproteins in the plant, including arabinogalactans, inulin, and heteroxylans, possess immunostimulatory and anti-inflammatory activities that aid in immune modulation, thus reducing inflammation often experienced during illness episodes [14,15,16].

Plant-derived compounds have also been used to treat diarrhoea, a major global health issue. Several scientific studies have found that herbal extracts act as antisecretory agents, have antiperistaltic effects, and antimicrobial and antispasmodic properties. Apigenin and friedelin have been identified as antidiarrhoeal agents because of their antisecretory and antimotility activity [17].

*Arctostaphylos uva-ursi* and *Vaccinium macrocarpon* have been used to treat urinary tract infections, and the essential oils from *Allium sativum*, *Melaleuca alternifolia*, and *Melissa officinalis* have been extensively used to treat respiratory, GI, urinary, and skin infections [18].

The examples above show that plant-derived phytochemicals may play a role in traditional medicine, offering potential remedies for various health conditions. With the integration of modern and traditional medical systems and the exploration of the world’s under-explored biodiversity, there is immense potential to discover novel phytochemicals and drug leads.

### 1.2. Examples of Approved Commercial Phytochemical Drugs

**Figure 1:**
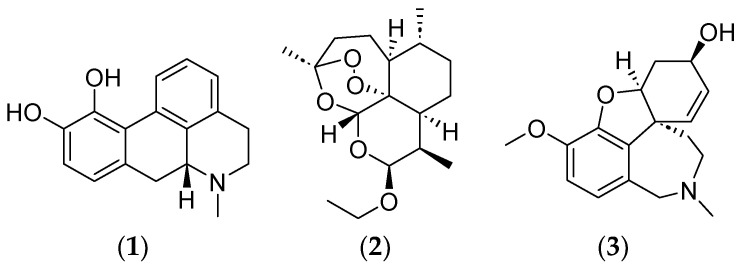
Phytochemicals used as drugs. Apomorphine (**1**), arteether (**2**) and galantamine (**3**).

Several plant-derived drugs on the market have been developed to treat various diseases (Figure 1), e.g., apomorphine (**1**) is made semi-synthetically from morphine isolated from *Papaver somniferum* L. While initially investigated as a potential non-addictive morphine replacement, the pharmacology of **1** is distinct, acting as a dopamine receptor agonist and it is now approved for the treatment of Parkinson’s disease [19]. Arteether (**2**) is a semisynthetic drug derived from artemisinin from *Artemisia annua* and is used to treat malaria. Arteether is oil soluble, has a long elimination half-life, and is more stable than artemisinin [20]. Galantamine (**3**) is an Amaryllidaceae alkaloid from *Galanthus woronowii* and an acetylcholinesterase inhibitor used in Alzheimer’s treatments [18]. Tiotropium is a muscarinic receptor antagonist from *Atropa belladonna* that has been used to treat asthma and chronic obstructive pulmonary disease (COPD) [21]. Other examples include anthocran, cysticlean, and monoselect macropcarpon from *Vaccinium* spp., which are being used to treat urinary tract infections. GutGuard is a standard product that was derived from *Glycyrrhiza glabra* extract, and Parodontax is a product that was developed from *Commiphora myrrha*, *Echinacea purpurea*, *Krameria triandra*, and *Matricaria recutita* extracts. *Mentha arvensis*, *M. piperita*, and *Salvia officinalis* essential oils have all been used to treat oral infections [22].

**Figure 2:**
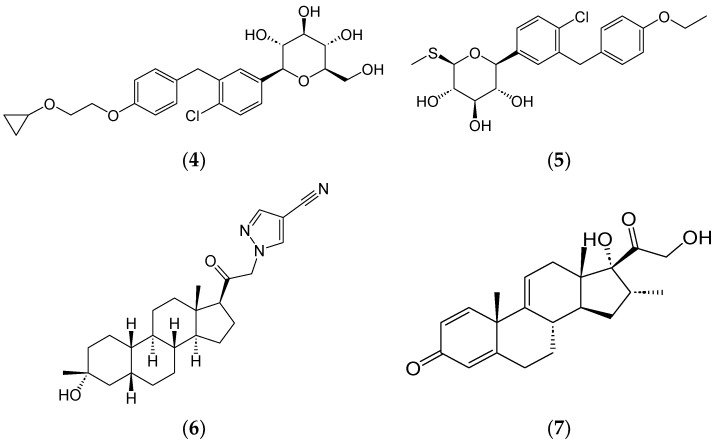
Synthetic small molecule drugs approved by the FDA in 2023. Bexagliflozin (Brenzavvy^TM^) (**4**), sotalgliflozin (Inpefa^TM^) (**5**), zuranolone (Zurzuvae^TM^) (**6**), and vamorolone (**7**) (Agamree^TM^).

Even in recent times, natural products play a role in drug development, with 6 of 53 new products approved by the FDA in 2023 having been inspired from natural products (Figure 2) [23]. Examples of small molecules approved include bexagliflozin (**4**) (Brenzavvy^TM^) and sotalgliflozin (**5**) (Inpefa^TM^), sodium-glucose co-transporter inhibitors that are synthetic analogues of the natural product phlorizin isolated initially from apple trees. Brenzavvy^TM^ has been authorized for glycemic control in adults with type 2 diabetes, and Inpefa^TM^ has been approved as a cardiovascular therapeutic. The synthetic steroids zuranolone (**6**) (Zurzuvae^TM^) and vamorolone (**7**) (Agamree^TM^) were respectively approved for the treatment of postpartum depression and Duchenne muscular dystrophy.

**Figure 3:**
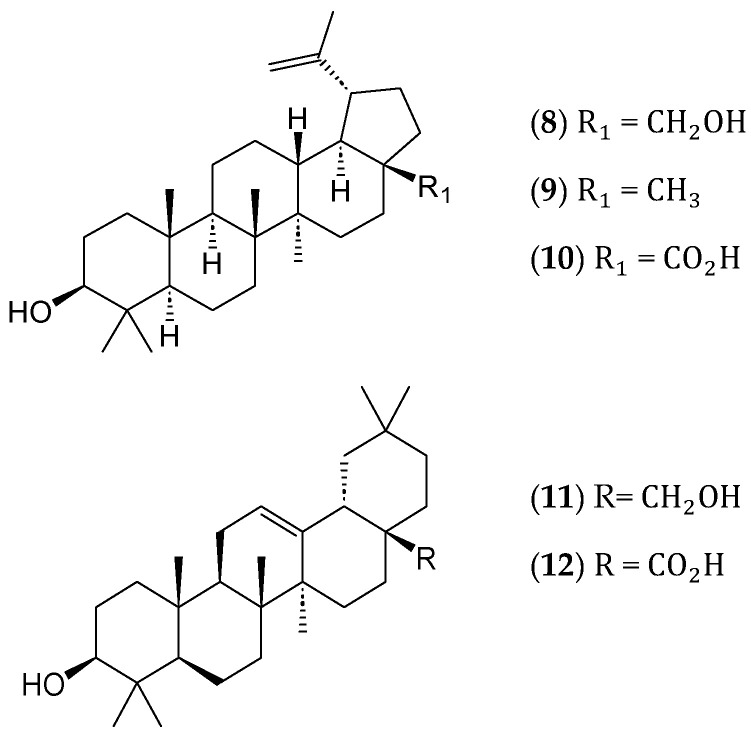
Phytochemicals found in Filsuvez^TM^ are composed of a mixture of pentacyclic triterpenes betulin (**8**), lupeol (**9**), betulinic acid (**10**), erythrodiol (**11**), and oleanolic acid (**12**) [23].

Filsuvez^TM^ is an extract of birch terpenoids that was approved in 2023. The topical gel consists of pentacyclic triterpenes (Figure 3), namely, betulin (**8**) (72–88%), lupeol (**9**) (2.4–5.7%), betulinic acid (**10**) (2.6–4.2%), erythrodiol (**11**) (0.5–1.2%), and oleanolic acid (**12**) (0.3–0.8%). The gel treats partial-thickness wounds with Junctional and Dystrophic Epidermolysis Bullosa (JEB and DEB). This is the first treatment approved for wounds associated with the rare disease JEB [23].

Natural products (NPs) or their derivatives contribute a substantial proportion of drugs that successfully progress through clinical trials to approval. A study by Domingo-Fernández et al. (2024) explored the features of natural products that contribute to their success. They analysed patent applications and found that synthetic compounds accounted for 77% of patents compared to 23% of NP and NP-derived patents. Next, they assessed clinical trial data, where they observed a steady increase in NP and NP-derived compounds going through clinical trial phases I to III (from approximately 35% in phase I to 45% in phase III), with an inverse trend observed in synthetics (from approximately 65% in phase I to 55% in phase III). Finally, they analysed *in vitro* and *in silico* toxicity studies that revealed that NPs and their derivatives were less toxic when compared to their synthetic counterparts. These observations offer valuable insights for successful NP-based drug development, which highlight the potential benefits of NPs and their derivatives [24].

## 2. Phytochemicals and Their Modern-Day Applications

### 2.1. Phytochemicals as Antivirals

Viral infections are one of the leading causes of morbidity and mortality. Examples of severe viral infections are Ebola, AIDS (acquired immunodeficiency syndrome), influenza, and SARS (severe acute respiratory syndrome) [25]. Viruses have several invasion mechanisms. Due to their genetic diversity, each virus has its unique biochemical configuration of surface molecules, which work like a lock and key, enabling viruses to enter hosts by accurately fitting the molecules on the surfaces of targets [26].

There is some evidence of antiviral potential of phytochemicals, particularly with tannins, yet little progress has been made in clinical analysis and product development. Preliminary studies suggest the feasibility of developing phytotherapeutics against viral infections [27,28,29,30]. For instance, saponins extracted from the bark of the soapbark tree (*Quillaja saponaria*) were successfully utilised as an adjuvant in the Pneumo-5 vaccine, offering potent protection against the bovine parainfluenza-3 virus [31,32].

**Figure 4:**
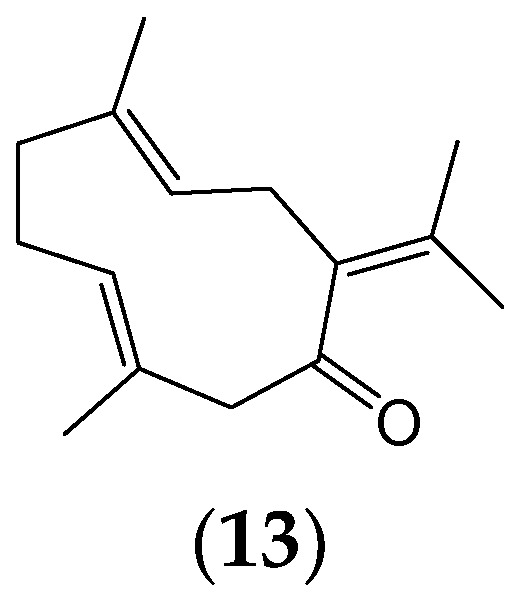
The sesquiterpene germacrone (**13**), synergistic with the antiviral agent oseltamivir.

Natural products may be selective antiviral agents [33], but their effectiveness can be limited by high cytotoxicity or low antiviral activity [34]. Further research on the anti-viral properties of phytochemicals will allow for the development of target-specific drug delivery systems. Very little knowledge exists of how phytochemicals interact with viruses or structures within the host cells. Therefore, there is a need to develop novel technologies and targeting strategies that can avoid cellular defences, transport phytochemicals to targeted intracellular sites, and release the phytochemicals in response to specific molecular signals [35]. Developing drug delivery systems, such as PEG-PLGA nanoparticles, can enhance their selectivity index and improve their protective properties against respiratory viruses [36,37]. Moreover, combining phytochemicals with established antiviral drugs may also enhance efficacy. For example, the sesquiterpene (Figure 4) germacrone (**13**), when paired with oseltamivir, demonstrated a synergistic effect in inhibiting influenza virus infection both in vitro and in vivo [38].

**Figure 5:**
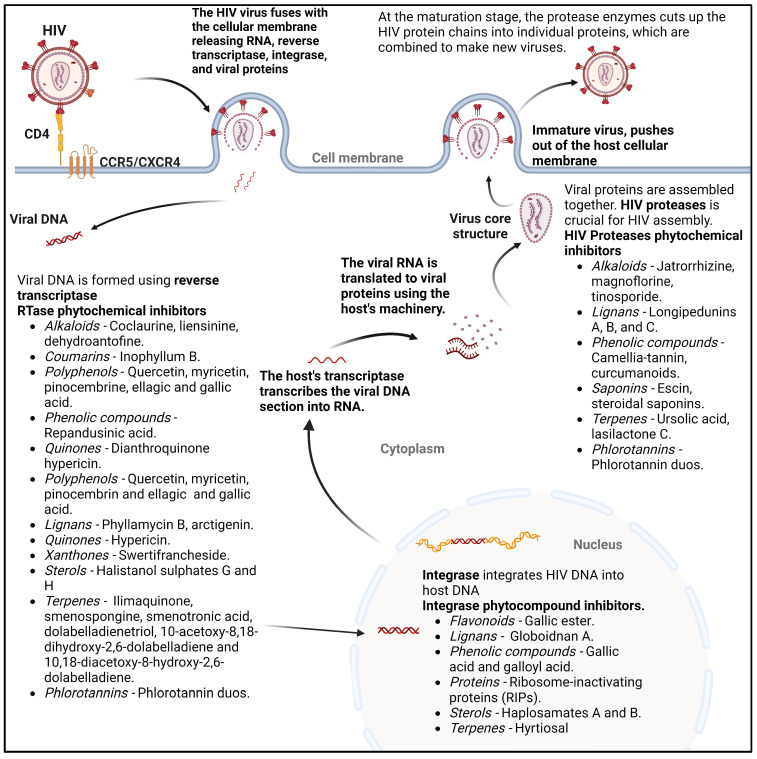
Phytochemicals showing anti-HIV potential adapted from [39]. Created with BioRender.com.

The Human Immunodeficiency Virus (HIV) is classified as a ‘balanced pathogen’. It persistently resides in the human body without immediately causing the catastrophic consequences observed with viruses such as COVID-19. It eventually progresses to terminal illness through ongoing replication, necessitating rigorous treatment for its eradication [39]. Several phytochemicals may control HIV using various mechanisms, as shown in Figure 5. One study showed that Patentiflorin A was more effective in suppressing HIV than azidothymidine (zidovudine) [40].

Plants were used extensively by local communities during the COVID-19 pandemic [41,42,43,44]. Medicinal plants may possess anti-inflammatory and anti-oxidative properties [45]. Several phytochemicals, such as capsaicin, gallic acid, naringin, psychotrine, and quercetin, have shown some antiviral properties targeting COVID-19 [46]. Another in silico study by Hafidul et al. 2020 revealed that ginger metabolites geraniol, gingerol, shogaol, zingerone, zingiberene, and zingiberenol might have potential antiviral properties that can reduce the virulence of SARS-CoV-2. The high binding energy of these natural products showed that they may bind to the Spike (S) protein and disrupt binding to the ACE2 receptor on the host cell phytochemicals, as well as inhibit the main protease (Mpro) necessary for the virus’s replication [47].

### 2.2. Phytochemicals in Cancer Combination Therapies

Several phytochemicals possess multi-targeted activity that simultaneously act on various biological pathways implicated in cancer [48,49]. Incorporating nature-derived substances in poly-cures may benefit overall treatment efficacy through synergism [1,50]. Additionally, evidence indicates that combining various phytochemicals could yield potent synergistic effects that boost overall treatment efficiency while hindering the emergence of drug resistance [1,51].

The emergence of chemo-resistance has resulted in the development of combination cancer therapy [52]. Combining multiple compounds can create a synergistic effect, amplifying their therapeutic benefits. Synergy may lead to greater efficacy while requiring lower dosages for individual components, reducing unfavourable side effects. The potential synergy between natural products and co-administered drugs could yield more significant clinical responses for patients suffering from varied illnesses or health conditions. By targeting multiple pathways simultaneously, natural products may have the ability to overcome resistance triggered by mutations and modifications in single targets.

Additionally, combining natural products with conventional drugs creates selective pressure on pathogens and cells or causes reduced mutation rates [53,54]. Optimising the pharmacokinetic profiles of combined drug therapies is critical for achieving maximal therapeutic benefits while minimising adverse drug reactions. Incorporating natural products into these therapeutic strategies offers a novel approach to improving overall efficacy [55,56,57]. Natural products within this framework are advantageous due to their multifaceted forms and inherent molecular diversity, which generate many pharmacological effects [58,59,60].

**Figure 6:**
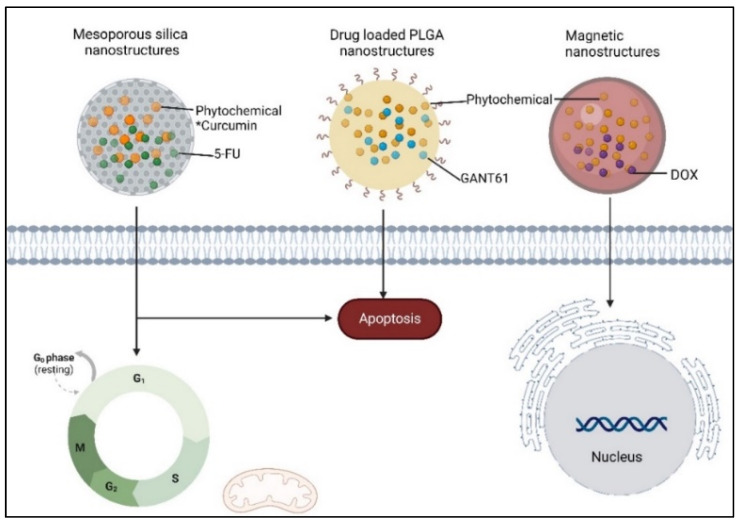
The co-delivery of phytochemicals in cancer therapy * adapted from [52], created with BioRender.com.

A study by Wang et al. (2022) demonstrated that nanoparticles can be used for co-delivery of these drugs in cancer therapy [61]. In recent studies, mesoporous nanoparticles were used to deliver 5-fluorouracil (5-FU), a chemotherapy drug used for chemotherapy and curcumin (Figure 6). This combination led to cell cycle arrest and apoptosis in laryngeal tumours (Hep-2 cells) [62].

**Figure 7:**
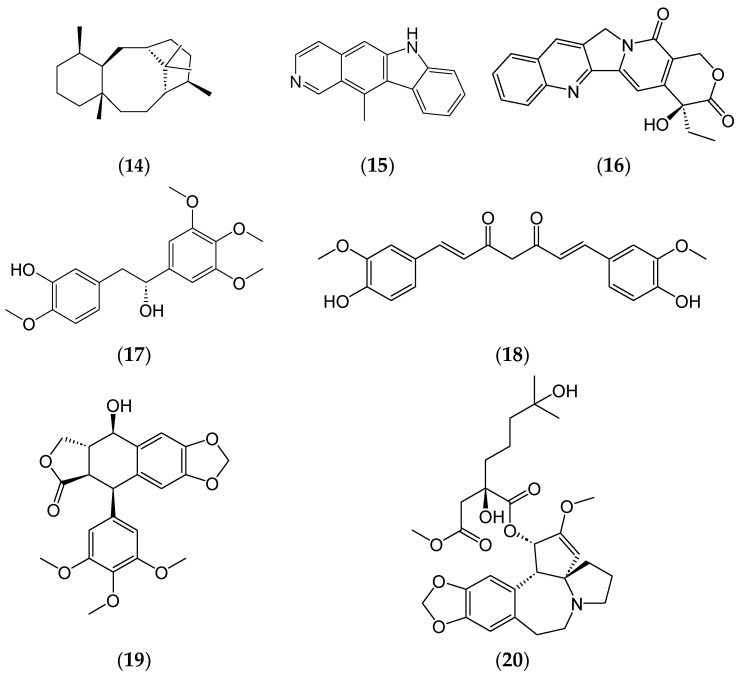
Chemical structures of taxane (**14**), ellipticine (**15**), camptothecin (**16**), combretastatin (**17**), curcumin (**18**), podophyllotoxin (**19**), homoharringtonine (**20**).

Drug repositioning and repurposing existing drugs for new therapeutic applications presents an opportunity for phytochemicals. Bioactive phytochemicals such as the taxanes (**14**), ellipticine (**15**), camptothecin (**16**), combretastatin (**17**), podophyllotoxin (**19**), homoharringtonine (**20**) (Figure 7), and others are reported for their potential anticancer effects on various neoplastic diseases [63].

Moreover, phytochemicals have been applied in cancer immunotherapy and vaccines and used as immune checkpoint inhibitors [64]. The FDA has confirmed the use of natural products and immunotherapeutic approaches in cancer treatment (described above). Moreover, the process of discovering cancer drugs has been accelerated by natural products. Approximately 47% of anti-tumour drugs have been reported to be derived from natural products [52,65,66,67,68,69,70,71,72]. Several studies have shown that natural compounds are capable of reducing estrogen receptor alpha (ER-α) levels, angiogenesis suppression proliferation and metastasis inhibition, apoptosis, and cell cycle arrest of breast tumours [52,73,74,75,76].

Moreover, phytochemicals targeting pathways like Hh, Notch, and Wnt/β-catenin and cancer stem cell resistance mechanisms show promise in reducing chemotherapy resistance. Therefore, it is crucial to assess plant-derived compounds’ safety, efficacy, and pharmacokinetic and pharmacodynamic properties [77].

### 2.3. Phytochemicals as Antimicrobials

The search for novel antibiotics should be accelerated as there are new microbial resistance determinants in bacteria, some of which have no effective remedies [78]. Microbial pathogens have developed self-defence machinery, which protects them against antimicrobial drugs, antibiotics, and pesticides (Figure 5). These mechanisms are active in pathogenic microbes, especially antibiotic-resistant phenotypes, ensuring their protection against a wide range of antibiotics [79,80].

Plants have been shown to possess antimicrobial activities, even in their crude form. The crude extracts or powders can further be purified to enhance potencies [81]. Several medicinal plant species are distributed across the African region and have been shown to possess some antimicrobial properties, e.g., *Hibiscus calyphyllus*, *Cassia abbreviata*, *Dicoma anomala*, *Securidosa longipendunculata*, and *Lippia javanica*, to name a few [82]. Phytochemicals can play an essential role in combatting antimicrobial resistance (AMR) and reducing the burden of infectious diseases. There is ongoing research in developing new antimicrobial therapies, which are currently supported by technological advancements in proteomics and metabolomics in Africa despite the economic challenges [82]. Phytochemicals may play an essential role in drug resistance since they are chemically diverse and possess a wide range of biological activities, which allows them to be used in complementary therapies [79]. They possess antimicrobial activities that can combat antimicrobial resistance when combined with multiple drugs with different mechanisms of action [1,83].

**Figure 8:**
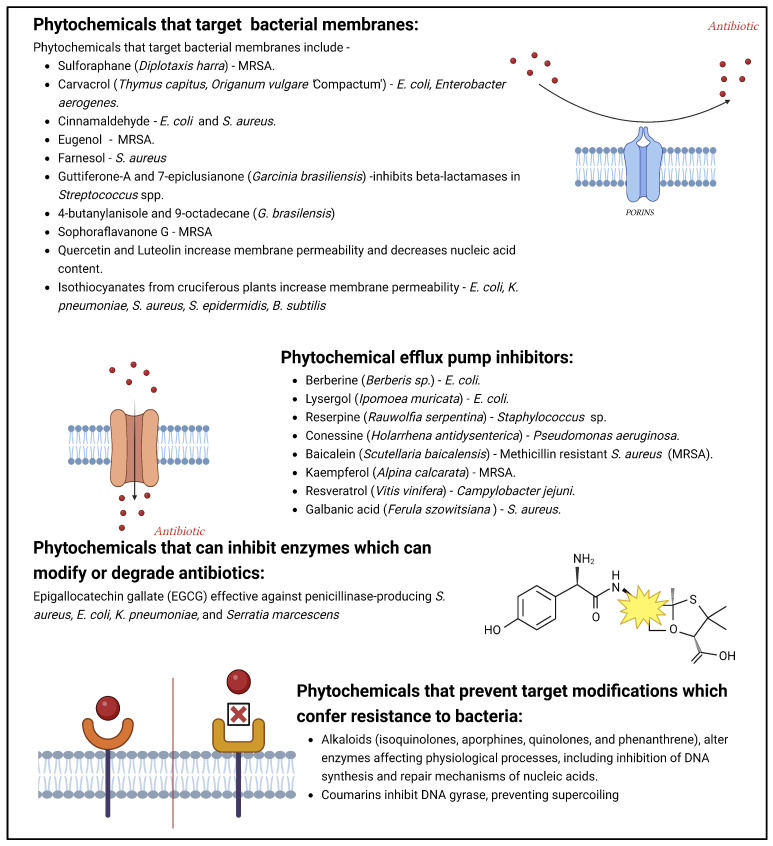
Phytochemicals and their mechanisms of action against drug resistance in microorganisms adapted from [22,79]. Created with BioRender.com.

The chemical diversity of phytochemicals offers a large repository for identifying novel drugs with distinctive modes of action (Figure 8). This heterogeneity allows these phytochemicals to home in on distinct cellular pathways and receptors, thus providing a better chance of discovering compounds that could potentially overcome drug resistance mechanisms encountered with current medications [1,84]. Phytochemicals can enhance antibiotic efficacy by disrupting bacterial cell walls, inhibiting efflux pumps, or modulating virulence factors (Figure 8).

## 3. Drug Discovery Approaches Using Phytochemicals

Synthetic compounds have dominated the field of medicinal chemistry [85]. Nevertheless, due to their diverse bioactivities, phytochemicals are increasingly considered promising alternatives for new drug development [1,84]. For instance, lead compounds can be obtained from phytochemicals such as alkaloids, terpenes, and flavonoids [1,86]. One key attribute supporting the importance of phytochemicals in drug discovery stems from their chemical diversity, broad spectrum of biological functions, and historical use within traditional medicinal practices [87]. As such, phytochemical screening is valuable for lead compound discovery efforts.

Drug discovery uses sophisticated techniques, including high-throughput screening, structure-based drug design, and computational methods [88] (Figure 6). Phytochemicals can be modified to enhance variables like drug effectiveness, resulting in an excellent resource pool to design new medical regimens specific to patients’ needs [89,90]. Though synthetic compounds have historically been a prominent source of drug candidates discovered via these approaches, natural products are also being explored. Despite this effort, identifying new drugs from natural sources remains daunting because of their complex structures and challenges in isolation and identification processes [91].

### 3.1. Traditional versus Modern Drug Discovery Methods

Pharmacological research has dedicated numerous years to seeking new compounds capable of efficiently treating different disorders. Exploring potential medications has included techniques such as rational drug design (producing synthetic molecules based on current drugs) or ethnopharmacology by adopting indigenous remedies. Additionally, using naturally occurring substances extracted from plants or animals (a natural-product-based strategy) has been utilised in several studies [1,92]. Although these methods resulted in positive effects in some situations, they demanded substantial effort and took significant periods for discovery.

Thanks to advances in structural and computational biology, exploring phytochemicals’ potential applications in drug discovery has never been more promising. With unparalleled detail and accuracy surpassing traditional laboratory experiments, these cutting-edge techniques give researchers unprecedented insight into biological processes, which is invaluable towards finding new treatments for the numerous maladies weighing heavily on humanity [1,93]. Despite this, identifying, designing, and testing promising drugs still presents formidable challenges that must be overcome.

#### 3.1.1. Traditional Drug Discovery Methods

Serendipitous events have played a crucial role in discovering life-saving medications in drug discovery. A great example is the chance discovery of penicillin by Alexander Fleming in 1928 when his bacterial culture was accidentally contaminated [94]. The discovery of ivermectin, an antiparasitic drug, resulted from a serendipitous collaboration between Satoshi Ōmura, who isolated the bacterium *Streptomyces avermitilis* from a soil sample in Japan, and William Campbell, who discovered its potential against parasites. This collaboration led to the development of ivermectin from avermectins. Although the drug was aimed at combating animal parasites, ivermectin was later approved for the treatment of human diseases like onchocerciasis and lymphatic filariasis. The discovery exemplifies the role of interdisciplinary research and the role of serendipity in medical breakthroughs. Ōmura and Campbell received the Nobel Prize in Physiology or Medicine in 2015 for their contributions [95].

In 1957, Kline et al. presented their findings on the therapeutic effect of iproniazid, a monoamine oxidase inhibitor, on depression at a regional meeting of the American Psychiatric Association in Syracuse, New York [96]. Iproniazid was synthesised in 1951 by Herbert Fox at Roche laboratories in Nutley, New Jersey (USA) for the chemotherapy of tuberculosis. However, in 1952, using iproniazid in tubercular patients, Orcnstein, Robitzek, and Sclikoff discovered that the drug produced euphoric behaviour in some patients. This unexpected observation, later confirmed by Zeller, led to further research, establishing Iproniazid as one of the first antidepressants [97].

Another serendipitous discovery is Khellin, a natural product derived from the plant *Ammi visnaga*. Traditionally used in Egypt for expelling renal calculi, researchers exploring its potential effects on smooth muscle discovered its vasodilating properties, which led to its application in treating angina pectoris [98]. Apomorphine’s use in Parkinson’s disease was also by chance. Apomorphine was developed as a non-addictive morphine replacement. Its pharmacological profile turned out to be distinct from morphine, exhibiting dopamine agonist activity that proved to be effective in managing Parkinson’s disease symptoms. This example highlights the importance of exploring the full range of biological activities of compounds, even those developed for entirely different purposes [99,100,101].

Although such occurrences can be unpredictable and unreliable for systematically identifying new drugs, ethnopharmacology’s study of traditional medicines and plant-based treatments has provided another promising avenue for innovation. Natural product screening is another method used to investigate various organic samples, including plants and microbes, along with defined criteria consistently uncovering novel biologically active molecules targeting multiple medical conditions across oncology, their diagnosis and treatment, and various bacterial/viral infections of multiple organ systems. Natural product (NP) screening normally involves a large library of extracts extracted from natural sources such as bacteria or plants. The extraction method significantly influences the type of compounds obtained. For example, more polar solvents yield more polar compounds in the crude extract. Therefore, to increase diversity, several solvents of varying polarities are often used. Once an extract with promising pharmacological activity is identified, it goes through successive bioactivity-guided fractionations until the pure bioactive compounds are isolated. There are several limitations associated with this method, for example, as some source organisms are non-culturable and in some instances some cease to produce NPs outside their natural habitat. However, to overcome these challenges, several new techniques have been developed. Examples include in situ analysis, NP synthesis induction, and heterologous expression of biosynthetic genes. Another common challenge is that the crude extracts may contain known NPs, NPs that are not drug-like, or inadequate quantities of NPs for characterization. This challenge can be addressed by developing methods for dereplication, extraction, and pre-fractionation [1].

Despite its effectiveness, conventional bioactivity-guided fractionation and isolation is a time-consuming process that may only sometimes lead to discovery of new compounds [1,102]. For instance, this method is currently being used to identify bioactive molecules from Traditional Chinese Medicines [103]. Moreover, library sizes have drastically increased, and traditional screening methods are no longer effective compared to virtual screening. For example, 1.2 billion non-covalent lead-like molecules and 6.5 million electrophiles were docked against the main viral protease (*MPro*). From these, 29 non-covalent and 11 covalent inhibitors were identified as potential inhibitors [104].

While conventional screening methods may provide empirical evidence of compound activity through direct or physical testing, virtual screening methods offer faster, more cost-effective ways to eliminate or prioritise compounds for further research.

#### 3.1.2. Modern Drug Discovery Methods

The integration of molecular biology, biochemistry, and structural biology has ushered in a new era for drug design [105,106,107]. Rational drug design represents a contemporary approach grounded in an exhaustive understanding of the disease mechanism and the target molecule’s structure and function. Such intrinsic knowledge enables researchers to develop particular and potent therapeutic agents targeting particular interactions. However, creating these agents requires extensive research into the disease and the targeted molecule’s nature. It is widely regarded as one of modern medicine’s most innovative approaches [108].

**Figure 9:**
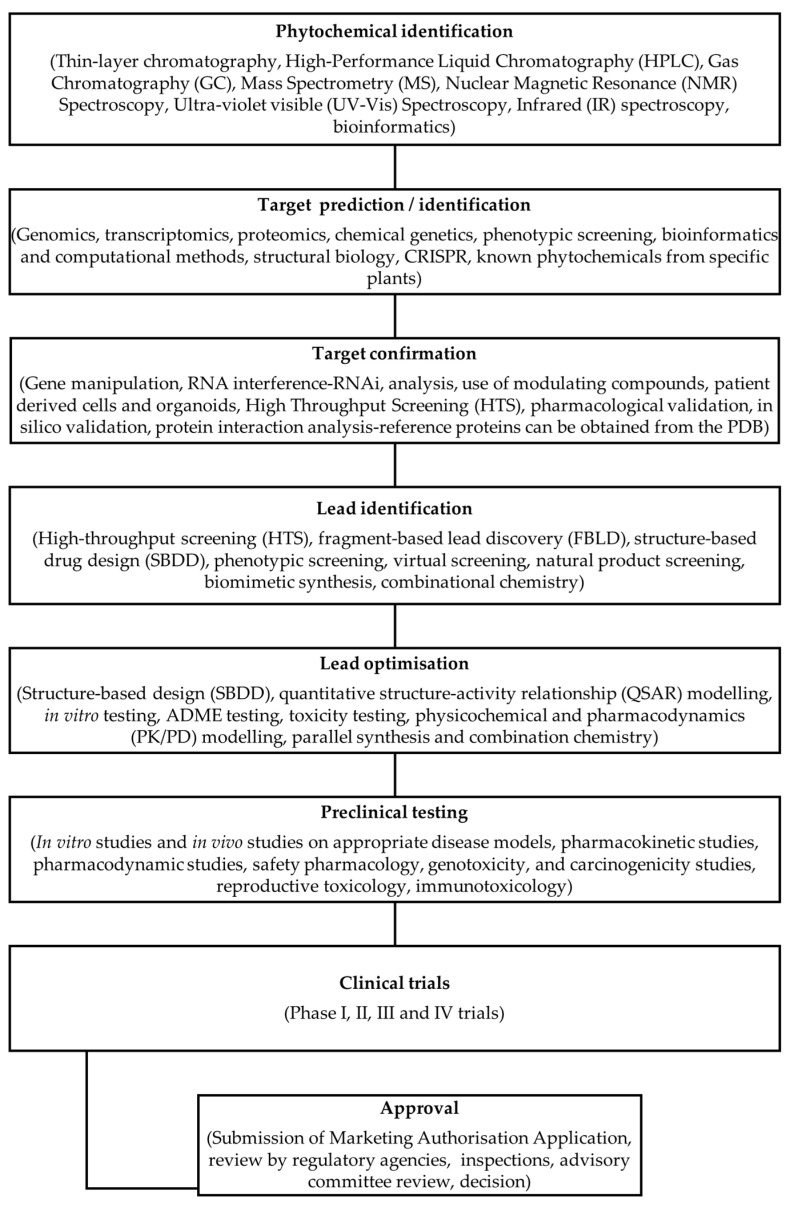
Sequential Stages in Phytochemical Drug Discovery and Development.

High-throughput screening (HTS) technology maximises efficiency while evaluating large libraries of compounds for their biological activity against specific targets or disease models within pharmaceutical research settings. HTS can be employed on several compound libraries, such as synthetic or natural product extracts, genome-scale gene knockouts, or RNA interference reagents (Figure 9). Although HTS yields rapid discoveries of active compounds, obstacles such as a lack of proper assay materials and potential inaccuracies may arise to limit its effectiveness [109,110].

Fortunately, computational tools like molecular modelling or docking enable researchers to expect interactions between generated molecules and their intended targets while also determining the chemical properties of these agents so they can assign priority levels before testing [111,112]. While computational methods have shown remarkable potential in accelerating drug discovery efforts, their success relies heavily on two primary factors: the quality and relevance of input data sets and the algorithms’ efficacy and precision [113,114,115].

Over the past years, several studies have explored the properties of phytochemicals as either adjuvants or inhibitors to enhance the potency of existing antibiotics, showing promising results for future medical applications. By employing computational techniques like virtual screening, molecular docking, QSAR modelling, and network pharmacology, scientists can quickly and more efficiently discover and enhance natural compounds with activity against drug-resistant targets (Figure 9) [1,29,116,117]. For example, Epigallocatechin Gallate (EGCG) found in green tea targets β-lactamases enzymes, which ae responsible for anti-biotic resistance in bacteria and efflux pumps [118,119]. Allicin is another natural compound found in garlic that targets bacterial efflux pumps and prevents biofilm formation [120].

Modern drug discovery methods provide insight into the mechanisms underlying phytochemical action towards drug resistance, thus adding to our understanding of such diseases. Advanced analytical methods that help isolate, identify, and characterise potential compounds have recently been applied. Furthermore, combining separation and detection methods through hyphenated approaches such as LC-MS and LC-NMR are efficient in streamlining compound identification (Figure 9) [121,122,123].

## 4. Computational Approaches to Identifying Potential Phytochemical Drugs

Computational approaches have emerged as an effective means of identifying and optimising phytochemical therapeutics. For instance, machine learning, virtual screening, molecular dynamics simulations, and molecular docking have previously been used to identify and modify the biological activity of phytochemicals (Figure 9) [124].

Virtual screening is a popular computational technique in drug discovery that can rapidly evaluate and prioritise compounds for experimental testing against a specific target or disease model [125,126,127]. Several approaches can be used, e.g., molecular descriptors and fingerprint-based similarity searching to ligand-based pharmacophore models or structure-based techniques. [128,129,130]. Virtual screening methods can be applied to large databases containing known phytochemicals or in-silico-generated libraries mimicking natural products [131]. This efficient technique manages large datasets and can reduce the number of compounds evaluated in biological assays [132].

### 4.1. Molecular Docking

Molecular docking has emerged as a game-changer in phytochemical drug discovery, offering a computational strategy to predict a phytochemical’s binding mode to its target protein(s) [133]. This tool is indispensable in selecting phytochemicals with high potential for further experimental investigation. There are numerous computational tools and algorithms available that have been developed. Examples of commonly used tools are AutoDock Vina, AutoDock GOLD, Discovery Studio, FRED, Glide, ICM, Surflex, MCDock, MOE-Dock, FlexX, DOCK, LeDock, rDock, Cdcker, LigandFit, and UCSF Dock [134]. Molecular docking has become indispensable in identifying molecular targets of nutraceuticals in the treatment of several diseases [124]

For instance, during the COVID-19 pandemic, molecular docking was instrumental in assessing and validating the ability of phytochemical ligands to interact with druggable targets for SARS-CoV-2 replication and pathogenesis [135] Among the predicted SARS-CoV-2 targets, the main protease or 3C-like protease (*3CLpro*) stood out as a significant druggable target due to its high conservation and the fatal impact its mutation would have on the virus [136]. A study by Tiwari et al. (2024) screened 408 phytochemicals from several plants that possess antiviral properties against the protein furin. Molecular docking revealed three compounds with good binding scores. Withanolide showed the lowest binding energy of −57.2 kcal/mol followed by robustaflavone and amentoflavone with a binding energy of −45.2 kcal/mol and −39.68 kcal/mol, respectively. Additionally, ADME analysis revealed drug-like properties for all three phytochemicals. Hence, they concluded that the three phytochemicals may have therapeutic potential for SARS-CoV-2 by targeting furin. Another study by Chouhan et al. (2023) also used computational methods to investigate microbially derived natural compounds against the *Mycobacterium tuberculosis* RpfB protein. They used structure-based virtual screening (SBVS), drug-likeness profiling, molecular docking, molecular dynamics simulation, and free-binding energy calculations [137].

**Figure 10:**
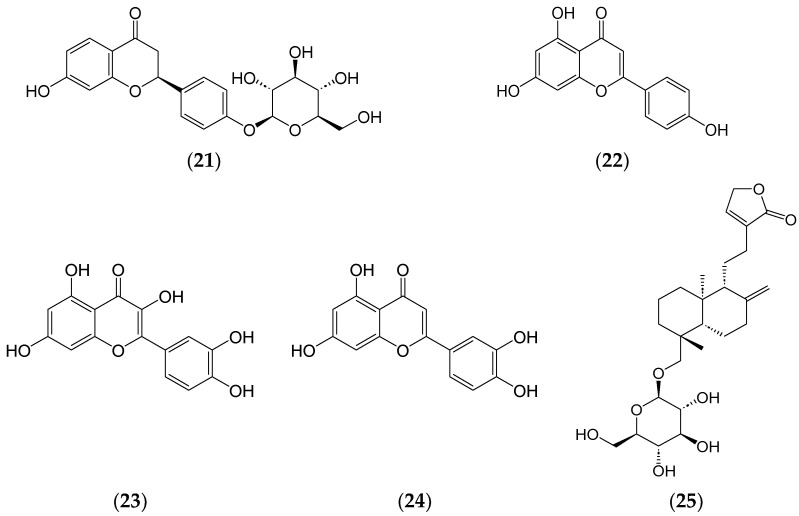
Chemical structures of liquiritin (**21**), apigenin (**22**), quercetin (**23**), luteolin (**24**), and neoandrographolide (**25**).

In another study, 43 drugs and 35 phytochemical candidates were selected for molecular docking studies based on their potential inhibitory effects towards the Spike glycoprotein of SARS-CoV-2. These candidates passed toxicity prediction and drug likeliness and demonstrated consistent docking to all the variants. Liquiritin (**21**) (a repurposed drug) and apigenin (**22**) (a phytochemical) (Figure 10) emerged as top contenders based on docking score, ADMET analysis, and drug likeliness profiles. However, in vitro and in vivo studies are yet to be carried out to validate its potency [138].

Additionally, other phytochemicals, such as phenolics and terpenoids, have shown potential as leads, including quercetin (**23**), luteolin (**24**), and neoandrographolide (**25**) (Figure 10) that were identified as potential inhibitors of SARS-CoV-2 druggable protein targets. It was shown that their interaction could disrupt viral replication and pathogenesis [139].

**Figure 11:**
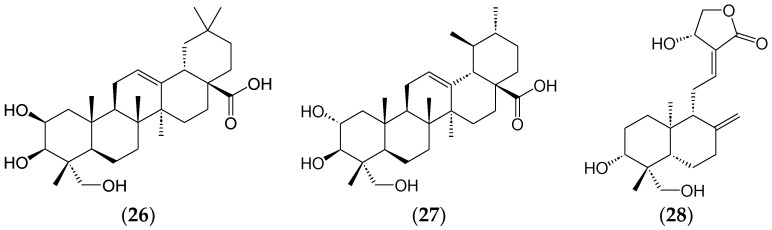
Bayogenin (**26**) asiatic acid (**27**), and andrographolide (**28**), potential leads against HK2.

Molecular docking has also been instrumental in discovering anticancer drugs from phytochemicals. For instance, in a study by Swargiary and Mani (2021), bayogenin (**26**), Asiatic acid (**27**), and andrographolide (**28**) (Figure 11) were revealed as the best lead compounds to target Hexokinase 2 (HK2) through molecular docking. Asiatic acid (**27**) also interacted with HK2, albeit less effectively than bayogenin and andrographolide. These compounds may be novel anticancer agents targeting HK2, pending further in vitro and in vivo experimental studies [140]. In another study on *Sauropus androgynus*, molecular docking and network pharmacology were employed to identify prime target genes and potential mechanisms, with AKT1, mTOR, AR, PPID, FKBP5, and NR3C1 being identified [141]. The PI3K-Akt signalling pathway, an essential regulatory node in various pathological processes, was significantly impacted. This study combined network pharmacology, molecular docking, and in vitro experiments to better understand the anticancer and anti-inflammatory molecular bioactivities of *S. androgynous* [141].

### 4.2. Molecular Dynamics

Molecular dynamics (MD) may also play an important role to refine docking or virtual screening results because biomolecules in the human body are dynamic, unlike the static conformations used in traditional structure-based drug design methods. Therefore, understanding the changing molecular structure of proteins can be critical. Molecular dynamics (MD) predicts biomolecule molecular and structural changes due to inter- and intramolecular forces, making it critical for drug discovery studies [132]. Several software tools can be used in MD simulation; for example, GROMACS 2024.2, AMBER 2024, and NAMD 3.0 are commonly used by computational scientist because of their robust computational algorithms and ability to handle large and complex molecular systems [142]. Moreover, molecular dynamic simulations can augment structure models by adding dynamics and atomic-scale movements [143]. Several studies have used MD simulations to study the interaction between flavonoids and G-quadruplex DNA. The use of MD simulations is vital in predicting the affinity of flavonoids for binding to G-quadruplex DNA, which plays an important role in cancer treatment [144,145]

### 4.3. Machine Learning and Artificial Intelligence

**Figure 12:**
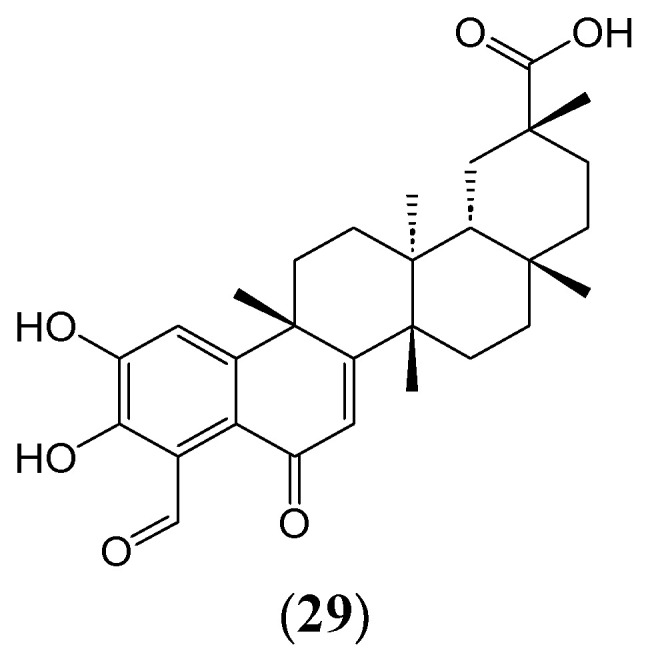
The phytochemical demethylzeylasteral (**29**) is a glucocorticoid receptor antagonist.

Artificial Intelligence and machine learning (ML) algorithms hold immense potential for improving our understanding of phytochemistry and its application to medical science. For example, there are several phytochemicals whose mechanisms of actions of action have not been elucidated. Understanding the mechanism-of-action (MoA) of phytochemicals and the prediction of potential drug targets plays an important role in small-molecule drug discovery. For example, a study by Trapotsi et al. (2021) compared bioactivity data from the escape database and cell morphology information from the Cell Painting Data to predict bioactivity data of compounds [146]. The same approach can be used to predict the bioactivities of phytochemicals in future studies. These methods offer convenient ways to explore how certain structural elements affect a compound’s qualities, such as bioactivity or biochemical behaviour. Researchers have provided several insights into phytochemical research by employing machine learning (ML) techniques like deep learning and network-based approaches [147]. These powerful computational tools allow researchers to analyse the intricate connections between phytochemical molecular characteristics and their biological properties, thereby identifying potential targets for therapy development. Moreover, machine learning offers new avenues for developing therapies based on particular phytochemicals to predict possible patient-specific reactions [148]. In one study, Shin et al. (2023) developed a workflow comprising two quantitative structure-activity relationship-based machine learning models to discover novel glucocorticoid receptor (GR)-antagonizing phytochemicals. The two models identified 65 phytochemicals that antagonised GR. They found that demethylzeylasteral (**29**) (Figure 12), a phytochemical of the *Tripterygium wilfordii* Radix, exhibited potent anti-obesity activity in vitro [149].

Another study evaluated a novel computational screening strategy that classified bioactive compounds and plants in semantic space generated by word embedding algorithms. The classifier showed good performance in binary (presence/absence of bioactivity) classification for both phytochemicals and plant genera, and this strategy led to the discovery of antimicrobial activity of several essential oils from *Cinnamomum sieboldii* and *Lindera triloba* against *S. aureus*. The results validated machine-learning classification in semantic space and showed that this approach can be helpful in exploring bioactive plant extracts [150].

García-Pérez et al. (2020) combined plant in vitro culture with neuro-fuzzy logic to characterise and optimise experimental conditions to produce phenolic compounds in the *Bryophyllum* spp. plant under nutritional stress. The algorithms could learn from experimental observations and construct a model with prediction abilities to characterise flavonoid content, total phenolic content, and radical-scavenging activity. They also proposed the combination of two cutting-edge methodologies involving plant in vitro culture and artificial intelligence-based tools to identify the phytochemical potential of under-exploited medicinal plants [151]. Artificial intelligence–based approaches such as machine learning have great potential for improving the bio-relevance of in vitro biological assays [152].

## 5. Phytochemical Limitations

Despite several advantages of using phytochemicals to develop drugs, several challenges can affect the efficiency, safety, and practicality of developing new drugs. The first issue is complexity and variability of phytochemical compositions in plants, which can lead to inconsistencies in characterisation and isolation of phytochemicals [153]. Environmental factors such as climate, geographical location, and soil quality play an enormous role in the phytochemical profile of the plant, making standardisation difficult [154,155]. Second, pure compounds’ extraction and purification processes are time-consuming and sometimes very low yields are obtained, which might affect the ability to conduct extensive pharmacological studies and further development [156,157]. Addtionally, the structures of several phytochemicals are very complex, which may pose difficulty when the structure needs to be modified or optimised.

Several phytochemicals have low bioavailability due to their poor solubility and stability characteristics. However, this challenge can be overcome by the developing drug delivery systems [158]. Lastly, safety and toxicity concerns pose another limitation since some phytochemicals might have adverse effects. Thus, comprehensive toxicological evaluations need to be carried out [159]. However, despite these challenges, ongoing research and technological advancements are gradually overcoming these challenges, enabling more effective use of phytochemicals in drug discovery.

## 6. Conclusions

Using phytochemicals as a basis for drug discovery is a promising avenue for creating novel therapeutic drugs. Moreover, coupled with current technologies, phytochemicals can be harnessed and applied in drug discovery processes. For example, optimising high-throughput screening and application of computational techniques can significantly streamline progress in creating effective therapeutics from phytochemicals. The use of emerging technologies and interdisciplinary research will help in maximising the potential benefits of phytochemicals in treating various diseases. By using or applying these cutting-edge tools, we can unlock new innovative therapeutics with far-reaching implications for patients worldwide.
